# Movement Disorder Patients with Depression Have Altered Corticostriatal Alpha-Beta Power Response to Reward and Loss

**DOI:** 10.1523/ENEURO.0008-26.2026

**Published:** 2026-07-09

**Authors:** Helen Qian, Graham W. Johnson, Natasha C. Hughes, Astoria Chao, Isabel C. Long, Danika L. Paulo, Zixiang Zhao, Deeptha Subramanian, Warren D. Taylor, Kaltra Dhima, Sarah K. Bick

**Affiliations:** ^1^Harvard Medical School, Boston, Massachusetts 02115; ^2^Department of Neurological Surgery, Vanderbilt University Medical Center, Nashville, Tennessee 37212; ^3^Vanderbilt University School of Medicine, Vanderbilt University, Nashville, Tennessee 37232; ^4^Department of Neurosurgery, Mayo Clinic, Rochester, Minnesota 55905; ^5^Department of Neurosurgery, Massachusetts General Hospital, Boston, Massachusetts 02114; ^6^Department of Neurosurgery, Henry Ford Hospital, Detroit, Michigan 48202; ^7^Department of Psychiatry and Behavioral Sciences, Vanderbilt University Medical Center, Nashville, Tennessee 37232; ^8^Geriatric Research, Education and Clinical Center (GRECC), Tennessee Valley Healthcare System Veterans Administration, Nashville, Tennessee 37212; ^9^Department of Neurology, Vanderbilt University Medical Center, Nashville, Tennessee 37232; ^10^Department of Psychiatry, UT Southwestern Medical Center, Dallas, Texas 75390; ^11^Department of Biomedical Engineering, Vanderbilt University, Nashville, Tennessee 37212

**Keywords:** corticostriatal, depression, essential tremor, intracranial EEG, Parkinson’s disease, reward

## Abstract

Depression is a common comorbidity in movement disorders such as Parkinson's disease (PD) and essential tremor (ET). Altered reward signaling contributes to core depression symptoms such as anhedonia, but the specific neural activity patterns underlying these processes and how they manifest in comorbid movement disorders are incompletely understood. Fourteen PD and 16 ET patients (22 male, 8 female) participated while undergoing deep brain stimulation surgery. Subjects completed a working memory task and received visual feedback about response accuracy while signals were recorded from traversed structures [caudate and/or dorsolateral prefrontal cortex (DLPFC)]. Preoperative Beck Depression Inventory-II (BDI-II) scores of ≥14 indicated elevated depression symptoms. Using cluster-based permutation testing, we identified time and frequency ranges in which oscillatory power significantly differed during reward versus loss feedback. We then used two-way ANOVAs and linear mixed effects models to assess how these power changes differed based on movement disorder and depression severity. Caudate and DLPFC alpha-beta (8–30 Hz) power increased during reward feedback. In both regions, this increase was attenuated in depressed subjects (caudate difference = −0.22, 95% CI = −0.32 to −0.13; DLPFC difference = −0.10, 95% CI = −0.16 to −0.045). BDI-II score was a negative predictor of reward- and loss-related corticostriatal alpha-beta power (caudate estimate = −0.014, 95% CI = −0.020 to −0.0078; DLPFC estimate = −0.0075, 95% CI = −0.012 to −0.0029). Specific to PD, depressed patients had greater decreases in DLPFC alpha-beta power following loss feedback than nondepressed patients (difference = −0.10, 95% CI = −0.17 to −0.027). Our findings suggest that altered corticostriatal alpha-beta power may contribute to reward dysfunction in depression in patients with movement disorders.

## Significance Statement

Depression is prevalent in both the general population and patients with movement disorders such as Parkinson's disease (PD) and essential tremor (ET). Imaging studies have suggested that patients with depression have altered reward processing, but our understanding of the neural activity underlying this effect and its manifestation in comorbid neurological disorders is limited. Using intracranial EEG recordings in movement disorder patients, we show that depression symptom severity predicts altered corticostriatal alpha-beta oscillatory power (8–30 Hz) reward signaling and depressed PD patients show heightened alpha-beta power response to loss feedback. Our findings implicate alpha-beta power attenuation within corticostriatal circuits as a potential therapeutic target for guiding novel treatment development for treatment-resistant depression and the comorbid depression symptoms of movement disorders.

## Introduction

Depression is one of the most prevalent psychiatric disorders (Steel et al., 2014; Conroy et al., 2020), and it is also a prevalent comorbidity in movement disorders such as Parkinson's disease (PD) and essential tremor (ET), with 35–50% of PD and ET patients experiencing clinically significant depression symptoms (Miller et al., 2007; Reijnders et al., 2008; Louis, 2010; Prange et al., 2022). In both PD and ET, depression symptoms may precede the onset of motor symptoms suggesting that depression may be a primary symptom of these disorders (Louis et al., 2007). The loss of dopaminergic neurons that underlies PD motor symptoms may also contribute to the development of depression by disrupting reward signaling in mesocorticolimbic systems and corticostriatal circuits (Marsh, 2013; Prange et al., 2022). Although depression is also common in ET, relatively little is understood about its pathophysiology. Currently, PD and ET patients with depression are treated with similar antidepressant medications and psychotherapies as those used for primary major depressive disorder (MDD; Marsh, 2013; Starkstein and Brockman, 2017; Prange et al., 2022). Unfortunately, up to 30% of patients with depression are resistant to standard medical treatments (Zhdanava et al., 2021). A better understanding of the neural activity changes underlying depression is needed to develop improved treatment options for MDD and patients experiencing depression as a comorbidity of another neurological disorder.

Anhedonia, characterized by inability to experience pleasure associated with rewarding experiences, is a core feature of depression. Depression and anhedonia are associated with altered reward signaling (Ubl et al., 2015). Dopaminergic neurons in the midbrain ventral tegmental area play an important role in reward signaling (Schultz et al., 1997) and project to multiple brain regions including the caudate, nucleus accumbens, and prefrontal cortex to propagate reward information to regions involved in learning, memory, and action selection (Keiflin and Janak, 2015). Patients with MDD have attenuated neural responses to reward receipt in multiple cortical and subcortical regions including caudate, nucleus accumbens, prefrontal cortex, and anterior cingulate cortex (Kumar et al., 2008; Ubl et al., 2015; Segarra et al., 2016; Yang et al., 2016; Taylor et al., 2022). Patients with MDD also have reductions in caudate volume correlated with severity of anhedonia and depression symptoms, supporting the importance of corticostriatal circuitry in depression (Pizzagalli et al., 2009).

PD patients also exhibit deficits in reward processing compared with healthy controls (Drew et al., 2020). These reward processing deficits in PD may be modulated by dopaminergic medications and have been linked to anhedonia, apathy, and issues with impulse control (Torta and Castelli, 2008; Timmer et al., 2017; Drew et al., 2020). Imaging studies show that PD patients have reduced striatal activation in response to reward (Martin-Soelch et al., 2001; Torta and Castelli, 2008), which has been attributed to nigrostriatal dopaminergic deficits. These striatal activation deficits have been shown to be especially prominent in PD patients with depression (Timmer et al., 2017). As such, dopaminergic deficits in PD may contribute to impaired reward signaling and depression symptoms in this patient population. The pathophysiology of depression in ET patients is less well understood.

Previous work has shown that beta (15–30 Hz) frequency neural oscillations in the caudate and DLPFC increase during reward-related feedback in human subjects (Hosseini and Holroyd, 2015; Bick et al., 2019; Paulo et al., 2023). In this study, we used intracranial recordings in subjects undergoing deep brain stimulation (DBS) surgery for movement disorders to evaluate how reward-related caudate and DLPFC oscillatory power changes may be altered by depression symptoms and how these effects may be modulated by PD and ET.

## Materials and Methods

### Subjects

Thirty patients (22 male, 8 female) undergoing DBS surgery participated in this study between 2021 and 2023. Subjects were undergoing bilateral subthalamic nucleus (STN), globus pallidus internus (GPI), or ventral intermediate (VIM) nucleus of the thalamus DBS to treat motor symptoms of PD or ET. Electrode trajectories were planned according to standard clinical considerations. Human subjects were recruited from Vanderbilt University Medical Center. All patients undergoing new DBS implantation with electrode trajectories contacting the caudate and/or DLPFC within the study period were eligible to participate in this study. This study was approved by the Vanderbilt University Medical Center Institutional Review Board and all subjects provided written informed consent. Subjects completed the Beck Depression Inventory-II (BDI-II) preoperatively to quantify current depression symptoms. We categorized subjects as depressed if their BDI-II score was ≥14 (Jones et al., 2015; Gerbasi et al., 2022).

### Surgery and local field potential recordings

Patients underwent bilateral DBS electrode implantation surgery under local anesthesia with microelectrode recordings to guide clinical electrode placement. Dopaminergic medications were held the night prior to surgery according to clinical protocol to facilitate intraoperative motor testing. Per our standard clinical protocol, a custom stereotactic platform (FHC) was mounted with bilateral microdrives, each of which held two or three microelectrodes, allowing for simultaneous bilateral recordings. Macro contacts on the microelectrode protective tube (FHC) allowed recording of local field potentials (LFPs). LFPs were recorded via the FHC Guideline 5 system with a sampling rate of 1,000 Hz.

**Figure 1.**
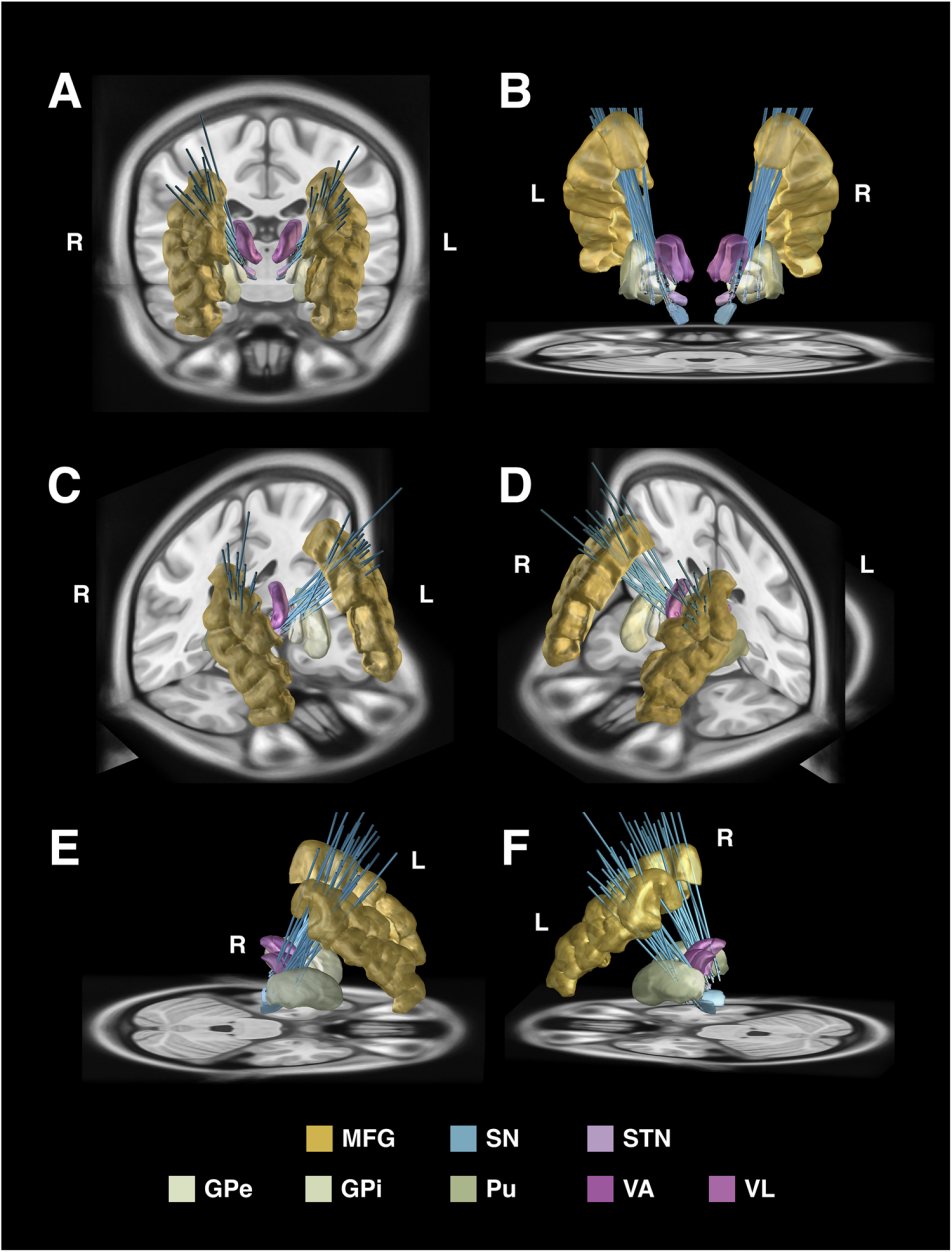
Cortical recording sites from DLPFC as demonstrated by electrode trajectories traversing the DLPFC. DBS electrode trajectories in patients and hemispheres with DLPFC recordings, visualized as extended solid electrodes to show the cortical entry points in the middle frontal gyrus where DLPFC recordings were performed. Electrode trajectories, cortical structures, and subcortical DBS target structures are visualized in the Montreal Neurological Institute standard space in anterior-posterior (***A***), posterior-anterior (***B***), three-quarter angle (***C***, ***D***), and sagittal (***E***, ***F***) views. R, right; L, left; DLPFC, dorsolateral prefrontal cortex; MFG, middle frontal gyrus; SN, substantia nigra; STN, subthalamic nucleus; GPe, globus pallidus externus; GPi, globus pallidus internus; Pu, putamen; VA, ventral anterior nucleus of the thalamus; VL, ventral lateral nucleus of the thalamus.

Microelectrodes were advanced through a rigid insertion tube along the planned clinical trajectory to the treatment target until the macro contact was within the structure to be recorded from for research purposes (caudate or DLPFC). For cortical recordings, the rigid insertion tubes and microelectrodes were advanced to just above the uncoagulated cortical surface, the microelectrode tip was advanced through the pia, and the protective tube with macro contact was advanced over this until the macro contact was just inside the cortex by visual inspection. The tube was held in place using the microdrive. LFP recordings were performed from the macro contact of the clinical microelectrodes via the FHC Guideline 5 system. Following research recordings, the rigid insertion tubes were advanced, and the microelectrodes and protective tubes were advanced to target depth to perform clinical recordings and stimulation per standard clinical protocol. Patients had up to three microelectrodes per side, allowing recordings from multiple channels within each structure, although only one brain region was recorded from per side. Twenty subjects had recording electrodes that traversed the caudate, and 26 subjects had electrodes that traversed the DLPFC for a total of 40 caudate and 79 DLPFC channels. To visualize where recordings were done within the DLPFC, DBS electrode trajectories that traversed the DLPFC were localized in Lead-DBS (Avants et al., 2008; Fonov et al., 2011; Horn et al., 2019). Postoperative CT was linearly coregistered to preoperative MRI using advanced normalization tools (Avants et al., 2011). Coregistrations were visually inspected, and a brain shift correction step was applied. DBS contacts were automatically prereconstructed using the PaCER method (Husch et al., 2018), and Atlas segmentations in this manuscript are defined by the Allen Human Reference Atlas (Ding et al., 2016). Electrode trajectories were visualized using the Lead group toolbox (Treu et al., 2020) as extended solid electrodes to show the cortical entry points in the middle frontal gyrus where DLPFC recordings were performed (Fig. 1).

### Behavioral task

**Figure 2.**
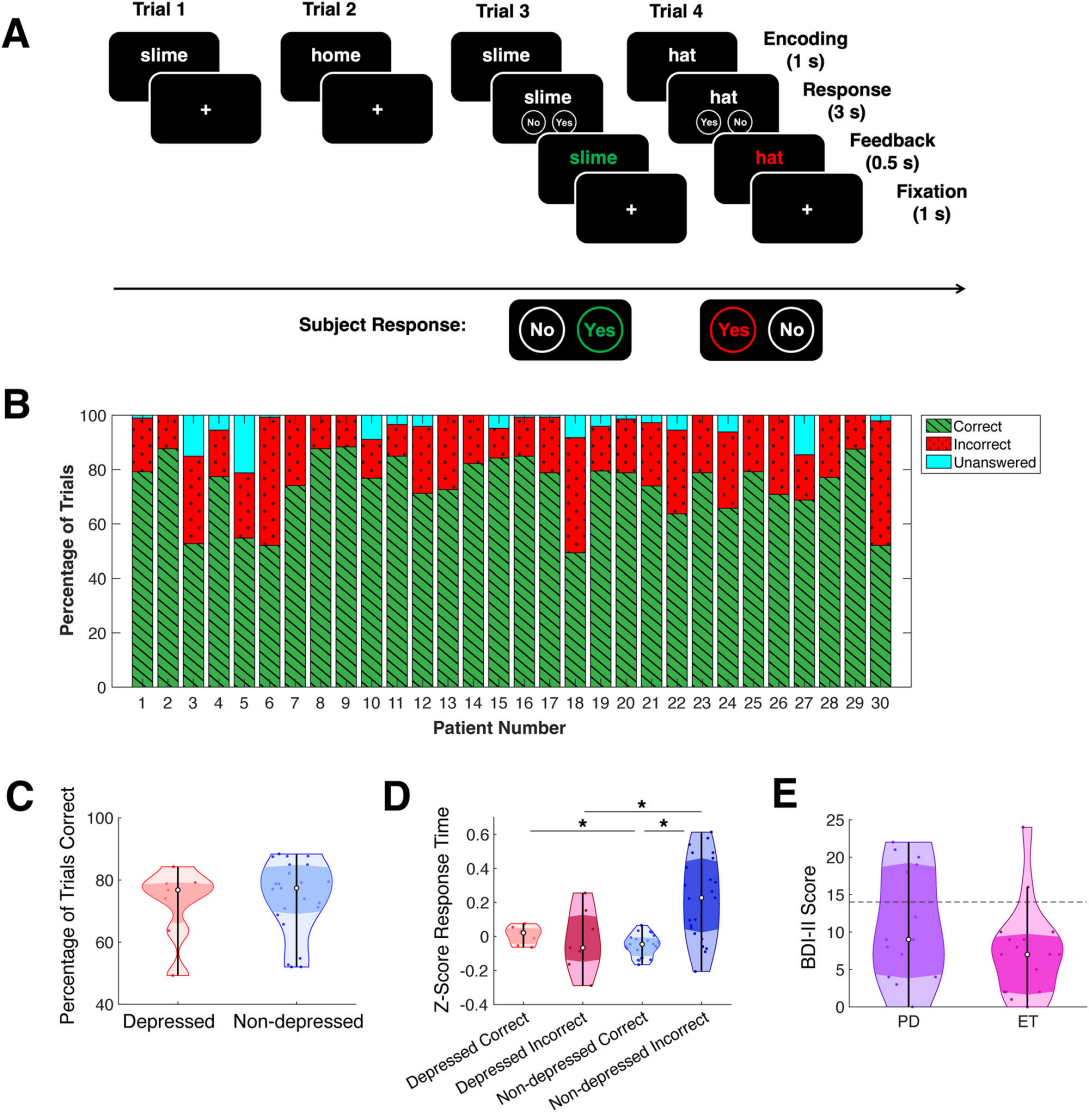
Task and patient performance. ***A***, Subjects participated in a two-back verbal working memory task (75 trials per block, 2 blocks per patient) as part of an ongoing study. Word stimuli were presented to the subject for 1 s, followed by a response cue prompting the subject to answer whether the current word matched the word presented two trials prior by pressing a button. The orientation of the buttons corresponding to Yes/No was randomly assigned on a trial-by-trial basis. Following response, visual feedback was presented for 0.5 s. Green represents correct response, and red represents incorrect response. ***B***, Task performance by subject: Percentage of trials that were correct (green), incorrect (red), and unanswered (cyan) per subject, averaged across blocks. ***C***, Violin plots comparing task accuracy for depressed and nondepressed patients. White circle indicates median, middle shaded region indicates interquartile range, and black line indicates the data minimum and maximum excluding outliers. There was no significant difference in performance between depressed and nondepressed patients. ***D***, Violin plots comparing *z*-scored response times between depressed and nondepressed patients. Average *z*-scored response time during correct trials was significantly slower for depressed (0.0077 ± 0.053 *z*-score) compared with nondepressed patients (−0.053 ± 0.064 *z*-score; difference = 0.060, 95% CI = 0.0060 to 0.11, *p* *=* 0.031), while depressed patients responded significantly faster for incorrect trials (−0.022 ± 0.19 *z*-score) than nondepressed patients (0.22 ± 0.24 *z*-score; difference = −0.25, 95% CI = −0.45 to −0.042, *p* *=* 0.020). There was a significant main effect of depression (*F*_(1,28)_ = 5.8, *p* = 0.023) and a significant interaction between depression status and trial accuracy on response time (*F*_(1,28)_ = 6.1, *p* = 0.020), with nondepressed patients responding significantly slower on incorrect trials compared with correct trials (difference = 0.28, 95% CI = 0.15 to 0.40, *p* = 7.7 × 10^−5^), while there was no difference between response time for correct and incorrect trials for depressed patients (difference = −0.030, 95% CI = −0.25 to 0.19, *p* = 0.78). ***E***, Violin plots comparing BDI-II scores in PD and ET patients. We categorized subjects as depressed if their BDI-II score was ≥14 (dashed line). BDI-II, Beck Depression Inventory-II; ET, essential tremor; PD, Parkinson's disease. *p < 0.05.

As part of a study evaluating LFP changes associated with cognitive impairment (Paulo et al., 2023), subjects participated in a two-back working memory task, during which they were visually presented with a series of words. For each word, a response cue appeared prompting the subject to press a button to indicate whether the current word matched the word presented two trials prior. Following response, subjects received visual feedback about response accuracy: the word turned green to indicate a correct response or red to indicate an incorrect response (Fig. 2*A*). Subjects completed two 75-trial blocks of this task. On average, completing one 75-trial block of the two-back task took patients 7.1 min. For the present study, we were interested in the feedback epoch and evaluating LFP changes associated with correct (rewarding) and incorrect (nonrewarding) responses.

### Neurophysiology and statistical analysis

LFP analysis was performed offline using MATLAB (MathWorks) and FieldTrip MATLAB toolbox (Oostenveld et al., 2011). Trials and/or channels with significant visual artifact were excluded. Data was notch filtered at 60 Hz, high-pass filtered at 1 Hz, and aligned to task events using digital event markers. Spectral power was calculated using Morlet wavelet time–frequency transformation in FieldTrip. For each task block, power was *z*-scored across all trials for each channel and frequency interval.

We first identified spectral power changes associated with reward using nonparametric cluster-based permutation testing (Maris and Oostenveld, 2007; Cecchi et al., n.d.) to identify time–frequency clusters of power during the 1,000 ms following feedback appearance that significantly differed between rewarded (correct) versus nonrewarded (incorrect) trials. First, we randomly shuffled response (power) and predictor (trial condition) pairs across trials 300 times at each time–frequency point for each channel and performed two-sided, two-sample Student's *t* tests to compare power between shuffled “correct” and “incorrect” trials for each channel. For each channel, we then randomly drew one of 300 shuffled permutations and performed two-sided, one-sample Student's *t* tests on the associated *t* statistics from the previous two-sample tests across recording sites to create a *t* statistic matrix for all time–frequency points. Contiguous time–frequency points with significant *t* values (*p* < 0.05) were grouped as clusters, with positive and negative *t* values being grouped separately. This was repeated 60,000 times. For each of the 60,000 randomizations, the maximum absolute value sum of *t* values within a cluster was used to compute a “null” distribution of effect size. The *p* value of each cluster derived from the nonshuffled regression estimates was calculated based on the proportion of clusters with higher statistics in the null distribution and then Bonferroni corrected based on the total number of clusters computed in the epoch of interest.

In both caudate and DLPFC, the most significant identified clusters spanned the alpha (8–15 Hz) to beta (15–30 Hz) frequency ranges. We therefore performed one-dimensional cluster-based permutation testing to identify time ranges in which averaged alpha-beta (8–30 Hz) power differed between rewarded and nonrewarded conditions in each region. For all subsequent analyses, we averaged alpha-beta power over these identified time ranges to compare between trial types and patient groups of interest.

We used Wilcoxon signed-rank tests to compare oscillatory power during reward and loss feedback to baseline power from the 2,000 ms intertrial interval. We then used a two-way repeated-measures ANOVA to assess the interaction between depression status and feedback type on averaged feedback power. For significant ANOVA results, we used the Tukey–Kramer post hoc test to evaluate pairwise comparisons. We performed this analysis across all subjects as well as separately for PD and ET patients.

We also assessed which channels in each brain region were significantly involved in our identified signals of higher alpha-beta power following reward feedback and lower alpha-beta following loss signals. For each channel, we performed two-tailed Wilcoxon signed-rank tests comparing alpha-beta power during the reward or loss feedback of each trial to alpha-beta power during the baseline period of that trial. For reward feedback comparisons, we designated a channel as significantly involved if alpha-beta power at that channel following reward was significantly higher than during baseline (*p* < 0.05). For loss feedback comparisons, we designated a channel as significantly involved if alpha-beta power at that channel following loss feedback was significantly lower than during baseline (*p* < 0.05).

To evaluate the effect of depression symptoms on reward signals in PD versus ET patients, we used two-way ANOVAs to assess the interaction between depression status and movement disorder diagnosis on average power during reward and loss feedback. We also fitted linear mixed effects models to evaluate the effect of BDI-II score, movement disorder diagnosis, and their interaction on reward-related power.

To evaluate whether task performance was associated with depression and neurophysiology signals, we used Pearson’s correlation to assess the relationships that reward-related power had with the percentage of correct trials and average response time for correct trials. To standardize response time comparisons, we *z*-scored response times across all trials per subject. We used Wilcoxon rank sum tests to evaluate the effect of depression status on task performance. We also performed a two-way repeated-measures ANOVA to examine the interaction between depression status and response accuracy on response time. When applicable, *p* values were Bonferroni corrected for multiple comparisons based on the number of brain regions analyzed (indicated by *p*_corr_).

### Code accessibility

The code/software described in the paper is freely available online at https://github.com/qhelen/MD-Depression-Reward. The code is available as Extended Data. Code was run on a 2019 MacBook Pro and the Sonoma 14.1 operating system.

10.1523/ENEURO.0008-26.2026.d1 Data Analysis Code The code/software described in the paper is freely available online at https://github.com/qhelen/MD-Depression-Reward. Download Data Analysis Code, ZIP file.

## Results

### Subjects and behavioral data

Fourteen subjects with PD and 16 with ET participated (Table 1, Extended Data Table 1-1). Five out of 14 (36%) PD patients and two out of 16 (13%) ET patients in our study cohort were categorized as depressed (Fig. 2*E*). Average working memory task performance was 73.8% correct, 22.5% incorrect, and 3.7% unanswered trials (Fig. 2*B*). Working memory accuracy did not differ between depressed and nondepressed patients (difference = 2.0%, 95% CI = −8.5 to 12.5%, *p* = 0.52, *z* = −0.64; Fig. 2*C*).

10.1523/ENEURO.0008-26.2026.t1-1 Table 1-1 **Patient demographic and disease-related information.** DLPFC = dorsolateral prefrontal cortex, ET = essential tremor, GPI = globus pallidus internus, PD = Parkinson’s disease, STN = subthalamic nucleus, VIM = ventral intermediate nucleus of the thalamus. Download Table 1-1, DOCX file.

**Table 1.** Patient demographic and clinical data summary

Group	Number of patients	Mean age (years)	Sex	DBS target	Mean % trials correct	Caudate channels	DLPFC channels
PD, nondepressed	9	62.6 ± 6.9	7M, 2F	5 STN, 4 GPI	77.1 ± 11.7	14	34
PD, Depressed	5	63.4 ± 8.2	5M	4 STN, 1 GPI	73.1 ± 13.8	9	15
ET, nondepressed	14	64.6 ± 10.8	9M, 5F	14 VIM	72.5 ± 12.0	17	24
ET, depressed	2	60 ± 7.1	1M, 1F	2 VIM	70.3 ± 9.3	0	6
Total	30	63.5 ± 8.9	22M, 8F	9 STN, 5 GPI, 16 VIM	73.8 ± 11.7	40	79

Detailed demographic and disease-related information per patient is given in Extended Data Table 1-1. DLPFC, dorsolateral prefrontal cortex; ET, essential tremor; GPI, globus pallidus internus; PD, Parkinson’s disease; STN, subthalamic nucleus; VIM, ventral intermediate nucleus of the thalamus.

Depression had a main effect on response time (*F*_(1,28)_ = 5.8, *p* = 0.023), with an interaction between depression status and accuracy (*F*_(1,28)_ = 6.1, *p* = 0.020). Nondepressed patients responded slower on incorrect trials compared with correct trials (difference = 0.28, 95% CI = 0.15 to 0.40, *p* = 7.7 × 10^−5^), while depressed patients showed no difference (difference = −0.030, 95% CI = −0.25 to 0.19, *p* = 0.78; Fig. 2*D*).

### Caudate and DLPFC oscillatory power during reward

**Figure 3.**
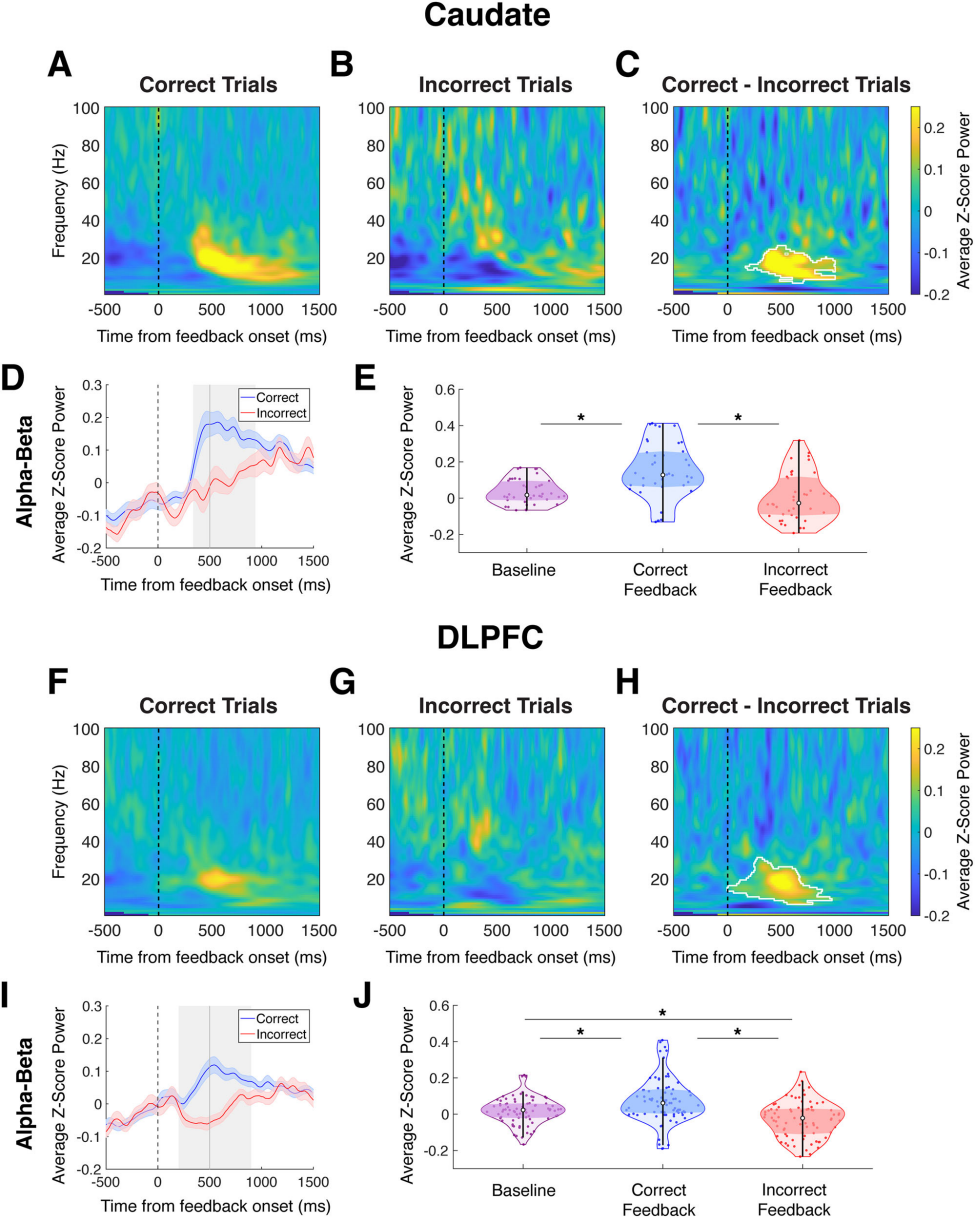
Caudate and DLPFC spectral power during reward and loss feedback. ***A–C***, ***F–H***, Spectrograms of *z*-scored power averaged over correct (***A***, ***F***), incorrect (***B***, ***G***), and the difference between correct and incorrect trials (***C***, ***H***) for caudate (***A–C***) and DLPFC (***F–H***). Dotted line indicates appearance of feedback. Time–frequency clusters with the greatest significant difference (*p*_corr_ < 0.002) between correct and incorrect trials are outlined in white. In both caudate and DLPFC, the most significant difference between correct and incorrect trials was in the alpha-beta frequency range. ***D***, ***I***, Time courses of average *z*-scored alpha-beta power during feedback for caudate (***D***) and DLPFC (***I***). Shading around the average line indicates standard error. Dotted line indicates feedback appearance and solid line indicates end of feedback presentation. Gray shaded boxes indicate time clusters where power was significantly different between correct and incorrect trials (*p*_corr_ = 6.7 × 10^−5^ for all comparisons). ***E***, ***J***, Violin plots comparing caudate (***E***) and DLPFC (***J***) alpha-beta power during baseline (2,000 ms intertrial period across all trials) with that following feedback for correct and incorrect trials. White circle indicates median, middle shaded region indicates interquartile range, and black line indicates the data minimum and maximum excluding outliers. In both the caudate and DLPFC, alpha-beta power following reward significantly increased from baseline (difference = 0.11, 95% CI = 0.053 to 0.18, *p*_corr_ = 2.0 × 10^−3^, *z* = −3.3 caudate; difference = 0.054, 95% CI = 0.016 to 0.091, *p*_corr_ = 8.8 × 10^−3^, *z* = −2.8 DLPFC). Following loss feedback, DLPFC alpha-beta power decreased from baseline (difference = −0.047, 95% CI = −0.079 to −0.015, *p*_corr_ = 1.5 × 10^−2^, *z* = 2.7). *p_corr_ < 0.05. DLPFC, dorsolateral prefrontal cortex.

On a group level, there was significantly greater 7–26 Hz power in the caudate (*p*_corr_ = 0.0014) and 7–31 Hz power in the DLPFC (*p*_corr_ = 0.0012) following feedback for rewarded compared with nonrewarded trials (Fig. 3*A*–*C*,*F*–*H*). Since the identified clusters from both structures encompassed the alpha and beta frequency bands, we focused our subsequent analysis on a combined alpha-beta (8–30 Hz) band. Averaged alpha-beta spectral power was significantly greater for rewarded compared with nonrewarded trials from 340 to 940 ms after feedback appearance in the caudate (*p*_corr_ = 6.7 × 10^−5^; Fig. 3*D*) and from 200 to 900 ms in the DLPFC (*p*_corr_ = 6.7 × 10^−5^; Fig. 3*I*).

To evaluate whether the observed power differences were associated with task-related processes, we compared alpha-beta power during feedback to baseline power. In both structures, alpha-beta power following reward significantly increased from baseline (caudate difference = 0.11, 95% CI = 0.053 to 0.18, *p*_corr_ = 2.0 × 10^−3^, *z* = −3.3; DLPFC difference = 0.054, 95% CI = 0.016 to 0.091, *p*_corr_ = 8.8 × 10^−3^, *z* = −2.8). Following loss, DLPFC alpha-beta power decreased from baseline (difference = −0.047, 95% CI = −0.079 to −0.015, *p*_corr_ = 1.5 × 10^−2^, *z* = 2.7) while caudate alpha-beta power did not change (difference = −0.033, 95% CI = −0.086 to 0.020, *p*_corr_ = 4.3 × 10^−1^, *z* = 1.2; Fig. 3*E*, *J*). Neither task performance nor response time correlated with reward-related alpha-beta power in the caudate (task performance *R* = 0.10, 95% CI = −0.35 to 0.52, *p*_corr_ > 1; response time *R* = −0.46, 95% CI = −0.75 to −0.025, *p*_corr_ = 0.080) or DLPFC (task performance *R* = 0.19, 95% CI = −0.22 to 0.54, *p*_corr_ = 0.72; response time *R* = −0.25, 95% CI = −0.58 to 0.16, *p*_corr_ = 0.45).

### Depression symptoms and reward-related alpha-beta power

**Figure 4.**
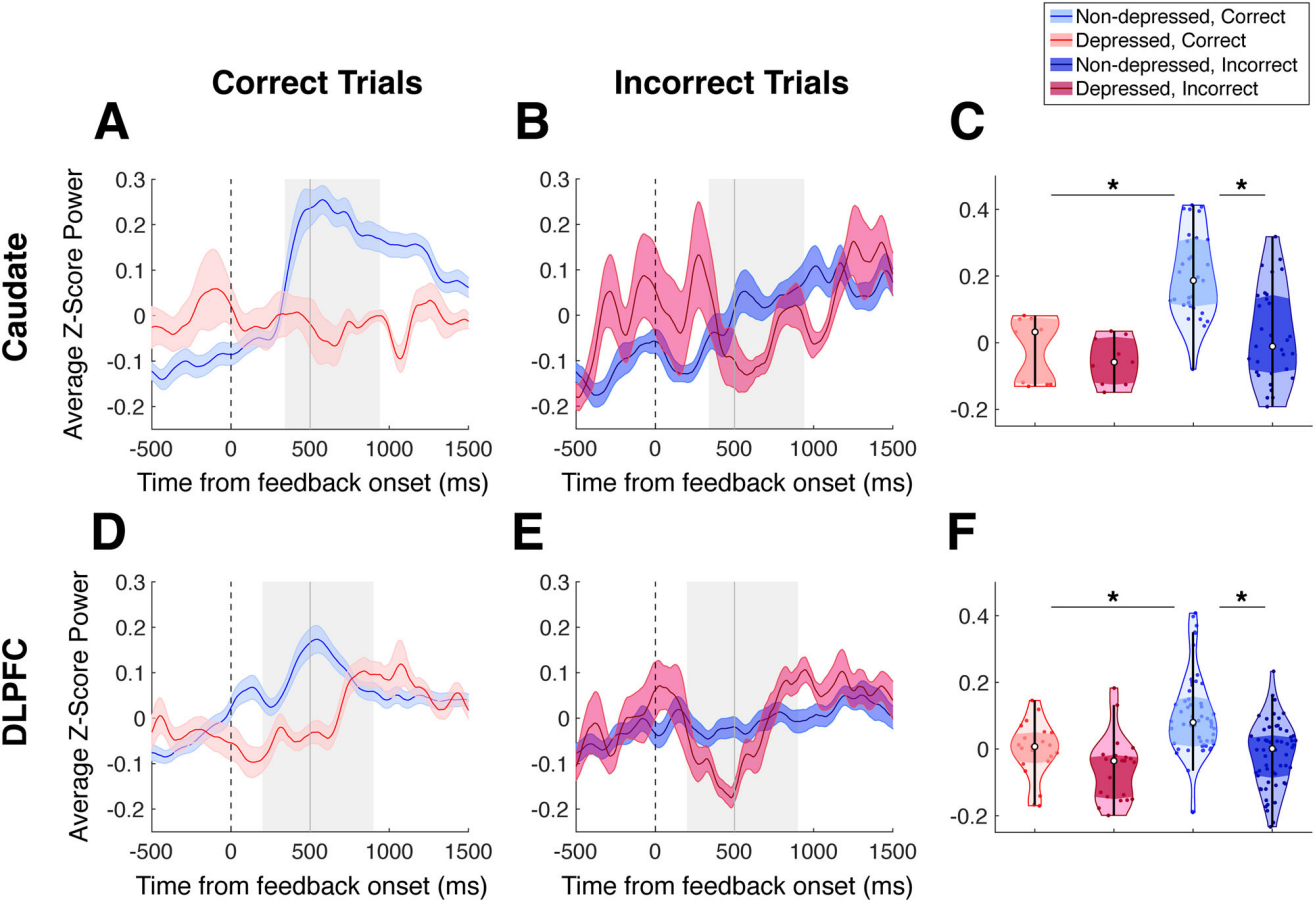
Impact of depression status on caudate and DLPFC alpha-beta power during reward and loss feedback in all subjects. ***A–E***, Time course of average *z*-scored alpha-beta power in the caudate (***A***, ***B***) and DLPFC (***D***, ***E***) during feedback for correct (***A***, ***D***) and incorrect (***B***, ***E***) trials for depressed and nondepressed patients. Shading around the average line indicates standard error. Dotted line indicates feedback onset and solid line indicates end of feedback presentation. Gray shaded boxes indicate time clusters where power was significantly different between reward and loss trials. ***C***, ***F***, Violin plots comparing average alpha-beta power following reward and loss feedback in caudate (***C***) and DLPFC (***F***) between depressed and nondepressed patients. Following reward feedback, depressed patients had significantly lower alpha-beta power (caudate difference = −0.22, 95% CI = −0.32 to −0.13, *p*_corr_ = 3.7 × 10^−5^; DLPFC difference = −0.10, 95% CI = −0.16 to −0.045, *p*_corr_ = 0.0014) compared with nondepressed subjects. Nondepressed patients had significantly higher alpha-beta power following reward compared with loss feedback (difference = 0.18, 95% CI = 0.15 to 0.22, *p*_corr_ = 2.1 × 10^−10^ caudate; difference = 0.12, 95% CI = 0.081 to 0.15, *p*_corr_ = 1.4 × 10^−8^ DLPFC), while there was no difference between rewarded and nonrewarded trials in depressed patients (caudate difference = 0.032, 95% CI = −0.033 to 0.096, *p*_corr_ = 0.66; DLPFC difference = 0.056, 95% CI = −0.0035 to 0.12, *p*_corr_ = 0.13). *p_corr_ < 0.05. DLPFC, dorsolateral prefrontal cortex.

We next evaluated how depression status impacted feedback-related alpha-beta power. We found a significant main effect of depression on alpha-beta power following reward and loss feedback in both structures (caudate *F*_(1,38)_ = 12, *p*_corr_ = 0.0028; DLPFC *F*_(1,77)_ = 12, *p*_corr_ = 0.0016). Depressed patients had significantly lower reward-related power (caudate difference = −0.22, 95% CI = −0.32 to −0.13, *p*_corr_ = 3.7 × 10^−5^; DLPFC difference = −0.10, 95% CI = −0.16 to −0.045, *p*_corr_ = 0.0014; Fig. 4*A*,*C*,*D*,*F*). Alpha-beta power following loss feedback appearance was not significantly different between depressed and nondepressed patients (caudate difference = −0.075, 95% CI = −0.17 to 0.023, *p*_corr_ = 0.26; DLPFC difference = −0.042, 95% CI = −0.091 to 0.0076, *p*_corr_ = 0.19; Fig. 4*B*,*C*,*E*,*F*). We also found a significant interaction effect between depression and reward/loss feedback type on alpha-beta power in the caudate (*F*_(1,38)_ = 17, *p*_corr_ = 0.00041) but not the DLPFC (*F*_(1,77)_ = 3.1, *p*_corr_ = 0.17). In both structures, nondepressed patients had significantly higher alpha-beta power following reward compared with loss feedback (caudate difference = 0.18, 95% CI = 0.15 to 0.22, *p*_corr_ = 2.1 × 10^−10^; DLPFC difference = 0.12, 95% CI = 0.081 to 0.15, *p*_corr_ = 1.4 × 10^−8^), while there was no difference between rewarded and loss trials in depressed patients (caudate difference = 0.032, 95% CI = −0.033 to 0.096, *p*_corr_ = 0.66; DLPFC difference = 0.056, 95% CI = −0.0035 to 0.12, *p*_corr_ = 0.13; Fig. 4*C*,*F*).

### Effect of PD and ET on depression modulation of reward signals

**Figure 5.**
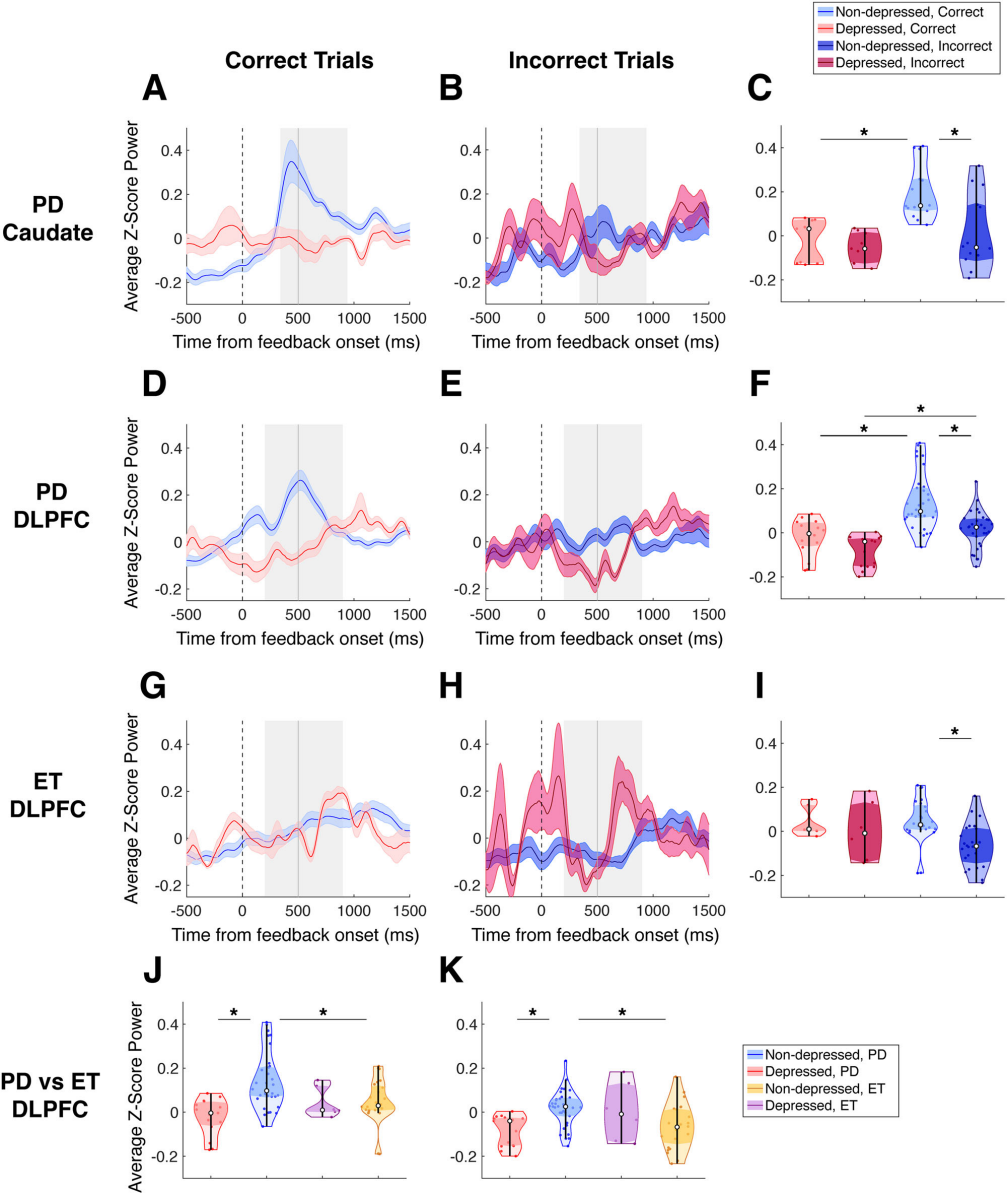
Impact of depression on alpha-beta power during reward and loss feedback in PD and ET subjects. ***A***, ***B***, ***D***, ***E***, ***G***, ***H***, Time course of average *z*-scored alpha-beta power in the caudate (***A***, ***B***) and the DLPFC (***D***, ***E***, ***G***, ***H***) during feedback for correct (***A***, ***D***, ***G***) and incorrect (***B***, ***E***, ***H***) trials for depressed and nondepressed patients with PD (***A***, ***B***, ***D***, ***E***) and ET (***G***, ***H***). Shading around the average line indicates standard error. Dotted line indicates feedback onset and solid line indicates end of feedback presentation. Gray shaded boxes indicate time clusters where power was significantly different between reward and loss trials. ***C***, ***F***, ***I***, Violin plots comparing average alpha-beta power following reward and loss feedback between depressed and nondepressed patients with PD (***C***, ***F***) and ET (***I***). Following reward feedback, caudate and DLPFC alpha power was significantly higher in nondepressed PD patients compared with depressed PD patients (caudate difference = 0.22, 95% CI = 0.12 to 0.32, *p*_corr_ = 0.00042; DLPFC difference = −0.16, 95% CI = −0.25 to −0.070, *p* = 6.01 × 10^−5^). Following loss feedback, depressed PD patients also had significantly lower DLPFC alpha-beta power (difference = −0.10, 95% CI = −0.17 to −0.027, *p* = 3.2 × 10^−3^ loss) compared with nondepressed PD subjects. There was no significant difference in DLPFC alpha-beta power between depressed and nondepressed ET patients following either reward (difference = 0.0037, 95% CI = −0.13 to 0.13, *p* = 1.0) or loss feedback (difference = −0.068, 95% CI = −0.18 to 0.040, *p* = 0.36). Caudate alpha-beta power in nondepressed PD patients was significantly higher following reward feedback compared with loss feedback (difference = 0.19, 95% CI = 0.13 to 0.25, *p*_corr_ = 2.3 × 10^−6^). In both PD and ET groups, DLPFC alpha-beta power was also significantly greater following reward compared with loss feedback in nondepressed patients (PD difference = 0.12, 95% CI = 0.071 to 0.17, *p*_corr_ = 2.6 × 10^−5^; ET difference = 0.11, 95% CI = 0.058 to 0.17, *p* = 2.1 × 10^−4^). ***J***, ***K***, Violin plots comparing average DLPFC alpha-beta power between PD and ET patients with and without depression following reward (***J***) and loss (***K***) feedback. Following reward feedback, alpha-beta power was significantly higher for nondepressed PD patients compared with depressed PD patients (difference = 0.16, 95% CI = 0.070 to 0.25, *p* = 6.01 × 10^−5^) and nondepressed ET patients (difference = 0.089, 95% CI = 0.014 to 0.17, *p* = 0.014). Following loss feedback, depressed PD patients (difference = −0.10, 95% CI = −0.17 to −0.027, *p* = 3.2 × 10^−3^) and nondepressed ET patients (difference = −0.081, 95% CI = −0.14 to −0.018, *p* = 0.0064) had significantly lower alpha-beta power than nondepressed PD patients. *p or *p*_corr_ < 0.05. DLPFC, dorsolateral prefrontal cortex; PD, Parkinson's disease; ET, essential tremor.

We also used two-way ANOVAs to evaluate whether the effect of depression symptoms on corticostriatal reward-related power differed between PD and ET patients. There were no depressed ET patients who had caudate recordings; therefore we could not evaluate the interaction between ET diagnosis and depression status on caudate reward-related alpha-beta power. We thus first evaluated the effect of depression status and correct versus incorrect feedback on caudate alpha-beta power in PD patients separately. We found significant main effects of depression (*F*_(1,21)_ = 8.0, *p*_corr_ = 0.020) and feedback type (*F*_(1,21)_ = 24, *p*_corr_ = 0.00014) on caudate alpha-beta power, as well as a significant interaction effect between depression status and feedback type (*F*_(1,21)_ = 12, *p*_corr_ = 0.0042). Nondepressed PD patients had significantly higher caudate alpha-beta power following reward compared with loss feedback (difference = 0.19, 95% CI = 0.13 to 0.25, *p*_corr_ = 2.3 × 10^−6^), while this was not significantly different in depressed patients (difference = 0.032, 95% CI = −0.041 to 0.10, *p*_corr_ = 0.75; Fig. 5*A*–*C*).

Our subsequent analysis focused on the interaction between movement disorder diagnosis and depression status on DLPFC reward-related alpha-beta power. In both PD and ET patients, feedback type had a significant main effect on alpha-beta power (PD *F*_(1,47)_ = 17, *p*_corr_ = 0.00033; ET *F*_(1,28)_ = 6.7, *p* = 1.5 × 10^−2^). For PD patients, depression has also had a significant effect (*F*_(1,47)_ = 38, *p*_corr_ = 2.7 × 10^−7^). For both movement disorders, nondepressed patients had significantly higher DLPFC alpha-beta power following reward compared with loss feedback (PD difference = 0.12, 95% CI = 0.071 to 0.17, *p*_corr_ = 2.6 × 10^−5^; ET difference = 0.11, 95% CI = 0.058 to 0.17, *p* = 2.1 × 10^−4^), while depressed patients did not (PD difference = 0.062, 95% CI = −0.012 to 0.14, *p*_corr_ = 0.2; ET difference = 0.040, 95% CI = −0.067 to 0.15, *p* = 0.45; Fig. 5*D*–*I*).

Following reward feedback, there was a significant main effect of depression status (*F*_(1,75)_ = 7.4, *p* *=* 0.0081) as well as a significant interaction effect between depression and movement disorder (*F*_(1,75)_ = 6.7, *p* *=* 0.011) on DLPFC alpha-beta power. Alpha-beta power following reward was significantly higher for nondepressed PD patients compared with depressed PD patients (difference = 0.16, 95% CI = 0.070 to 0.25, *p* = 6.01 × 10^−5^) as well as nondepressed ET patients (difference = 0.089, 95% CI = 0.014 to 0.17, *p* = 0.014; Fig. 5*D*,*F*,*J*). Reward-related DLPFC alpha-beta power did not differ between nondepressed and depressed ET patients (difference = 0.0037, 95% CI = −0.13 to 0.13, *p* = 1.0; Fig. 5*G*,*I*,*J*).

Following loss feedback, there was a significant interaction effect between depression status and movement disorder (*F*_(1,75)_ = 11, *p* *=* 0.0011) on DLPFC alpha-beta power but no significant main effect of depression status alone (*F*_(1,75)_ = 0.42, *p* *=* 0.52). Depressed PD patients had significantly lower alpha-beta power following loss compared with nondepressed PD patients (difference = −0.10, 95% CI = −0.17 to −0.027, *p* = 3.2 × 10^−3^), while nondepressed PD patients had significantly higher loss-related alpha-beta power than nondepressed ET patients (difference = 0.081, 95% CI = 0.018 to 0.14, *p* = 0.0064; Fig. 5*E*,*F*,*K*). There was no difference in loss-related alpha-beta power between depressed and nondepressed ET patients (difference = 0.068, 95% CI = −0.040 to 0.18, *p* = 0.36; Fig. 5*H*,*I*,*K*).

**Figure 6.**
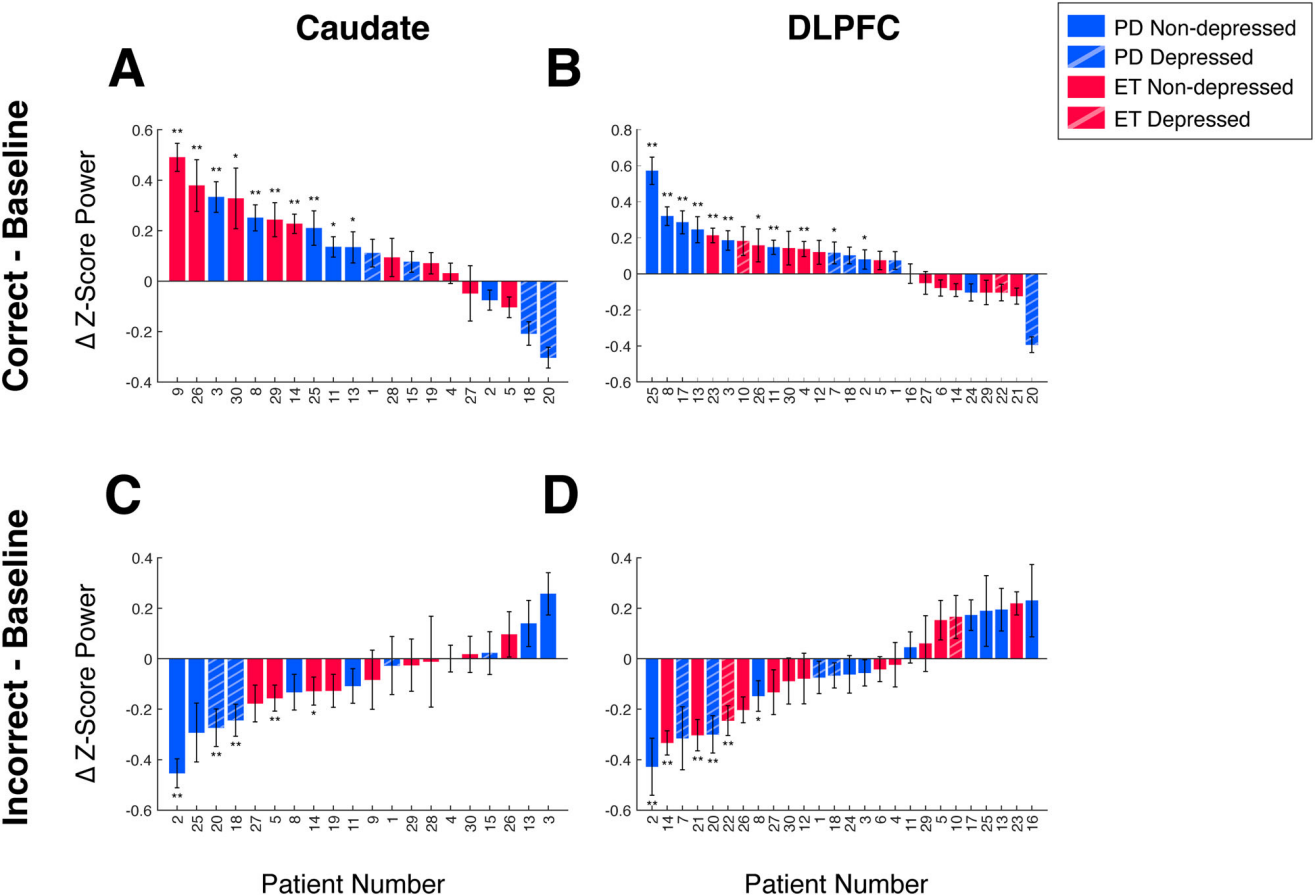
Subject-level changes in alpha-beta power following reward feedback. ***A***, ***B***, Bar graphs of the difference between average *z*-scored alpha-beta power following reward feedback compared with baseline for the channel in the caudate (***A***) or DLPFC (***B***) that had the most significant reward-related increase in power for that subject. Bars are ordered in descending order of the delta value of change in alpha-beta power. ***C***, ***D***, Bar graphs of the difference between average *z*-scored alpha-beta power following loss feedback compared with baseline for the channel in the caudate (***C***) or DLPFC (***D***) that had the most significant loss-related decrease in power for that subject. Bars are ordered in ascending order of the delta value of change in alpha-beta power. The number of caudate and DLPFC channels per subject with significantly higher alpha-beta power following reward or significantly lower alpha-beta power following loss feedback compared with baseline is given in Extended Data Figs. 6-1 and 6-2. **p < 0.01, *p < 0.05. DLPFC, dorsolateral prefrontal cortex; PD, Parkinson's disease; ET¸ essential tremor.

These results are consistent at the individual subject level. In our trial-level analysis of which caudate channels were significantly involved in reward signaling, we found that 10 channels from five PD patients and nine channels from five ET patients had significantly higher alpha-beta following reward feedback compared with baseline, while seven channels from three PD patients and two channels from two ET patients had significantly lower alpha-beta power following loss feedback compared with baseline. All patients with caudate channels that had significantly higher alpha-beta power following reward were categorized as nondepressed, while two PD patients with caudate channels that had significantly lower alpha-beta power following loss feedback were categorized as depressed (Fig. 6*A*,*C*; Extended Data Fig. 6-1). In the DLPFC, there were 18 channels from eight PD patients and four channels from three ET patients with significantly higher alpha-beta power following reward feedback compared with baseline. Of these, all patients except one PD patient were categorized as nondepressed. We also found six channels from three PD patients and seven channels from four ET patients that had significantly lower DLPFC alpha-beta power following loss feedback compared with baseline. Within these patients, one PD patient and one ET patient were categorized as depressed (Fig. 6*B*,*D*; Extended Data Fig. 6-2).

10.1523/ENEURO.0008-26.2026.f6-1 Figure 6-1 **Caudate channels significantly involved in reward signaling.** PD = Parkinson’s disease, ET = essential tremor. Download Figure 6-1, DOCX file.

10.1523/ENEURO.0008-26.2026.f6-2 Figure 6-2 **DLPFC channels significantly involved in reward signaling.** DLPFC = dorsolateral prefrontal cortex, PD = Parkinson’s disease, ET = essential tremor. Download Figure 6-2, DOCX file.

**Figure 7.**
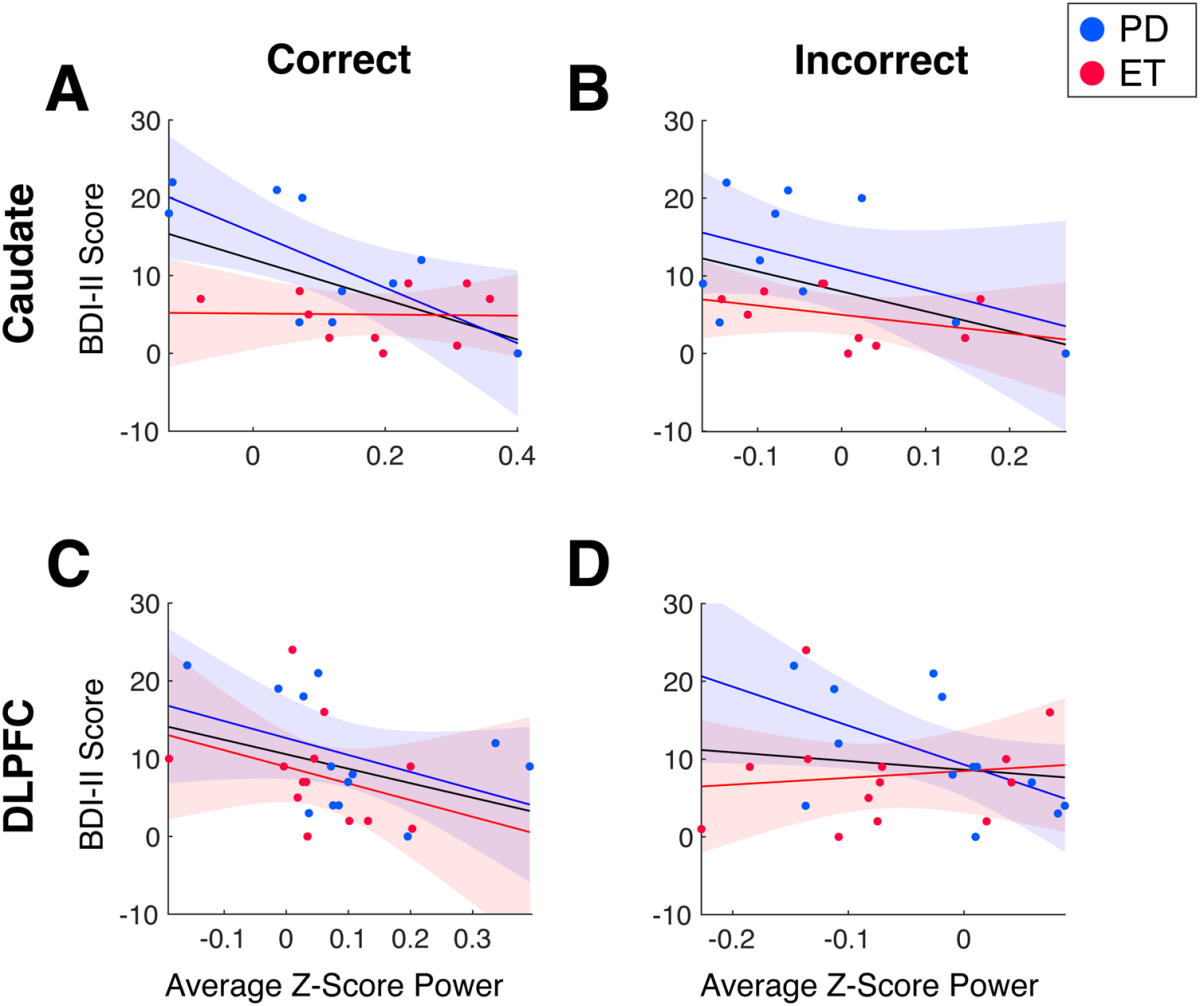
Relationship between BDI-II scores and alpha-beta power during reward and loss-related feedback. Average reward- (***A***, ***C***) and loss-related (***B***, ***D***) alpha-beta power per subject in the caudate (***A***, ***B***) and DLPFC (***C***, ***D***) plotted against BDI-II scores. Lines of best fit for the relationship between alpha-beta power and BDI-II score are shown for PD (blue), ET (red), and all patients (black). Shaded bands represent 95% confidence intervals for PD (blue) and ET (red) patients. Linear mixed effects model analyses of the relationship between BDI-II scores and reward- and loss-related power are given in Extended Data Figs. 7-1–7-4. Higher BDI-II score (more severe depression symptoms) was a significant predictor of lower caudate and DLPFC alpha-beta power following both reward (caudate estimate = −0.014, 95% CI = −0.020 to −0.0078, *p*_corr_ = 1.2 × 10^−4^; DLPFC estimate = −0.0075, 95% CI = −0.012 to −0.0029, *p*_corr_ = 3.5 × 10^−3^) and loss (caudate estimate = −0.0083, 95% CI = −0.015 to −0.0020, *p*_corr_ = 0.022; DLPFC estimate = −0.0076, 95% CI = −0.011 to −0.0041, *p*_corr_ = 0.00011) feedback. Movement disorder (estimate = −0.15, 95% CI = −0.22 to −0.079, *p*_corr_ = 0.00011) and the interaction effect between BDI-II score and movement disorder (estimate = 0.011, 95% CI = 0.0052 to 0.017, *p*_corr_ = 0.00082) were significant predictors for DLPFC alpha-beta power following loss feedback. DLPFC, dorsolateral prefrontal cortex; PD, Parkinson's disease; ET, essential tremor.

We also evaluated whether reward- and loss-related power was correlated with depression symptom severity as quantified by BDI-II score. Using a linear mixed effects model, we found a significant effect of BDI-II score on alpha-beta power following both reward (caudate estimate = −0.014, 95% CI = −0.020 to −0.0078, *p*_corr_ = 1.2 × 10^−4^; DLPFC estimate = −0.0075, 95% CI = −0.012 to −0.0029, *p*_corr_ = 3.5 × 10^−3^) and loss (caudate estimate = −0.0083, 95% CI = −0.015 to −0.0020, *p*_corr_ = 0.022; DLPFC estimate = −0.0076, 95% CI = −0.011 to −0.0041, *p*_corr_ = 0.00011) feedback, with higher BDI-II scores correlating with lower alpha-beta power. In the DLPFC, movement disorder (estimate = −0.15, 95% CI = −0.22 to −0.079, *p*_corr_ = 0.00011) and the interaction between BDI-II score and movement disorder (estimate = 0.011, 95% CI = 0.0052 to 0.017, *p*_corr_ = 0.00082) were significant predictors for alpha-beta power following loss but not reward feedback, while there was no impact of movement disorder on reward or loss-related power in caudate (Fig. 7; Extended Data Figs. 7-1–7-4).

10.1523/ENEURO.0008-26.2026.f7-1 Figure 7-1 **Linear mixed effects model results for caudate alpha-beta power during correct trials.** DF = degrees of freedom, CI = confidence interval. Download Figure 7-1, DOCX file.

10.1523/ENEURO.0008-26.2026.f7-2 Figure 7-2 **Linear mixed effects model results for DLPFC alpha-beta power during correct trials.** DF = degrees of freedom, CI = confidence interval. Download Figure 7-2, DOCX file.

10.1523/ENEURO.0008-26.2026.f7-3 Figure 7-3 **Linear mixed effects model results for caudate alpha-beta power during incorrect trials.** DF = degrees of freedom, CI = confidence interval. Download Figure 7-3, DOCX file.

10.1523/ENEURO.0008-26.2026.f7-4 Figure 7-4 **Linear mixed effects model results for DLPFC alpha-beta power during incorrect trials.** DF = degrees of freedom, CI = confidence interval. Download Figure 7-4, DOCX file.

## Discussion

In this study, we found that corticostriatal alpha-beta oscillatory power is elevated during reward and modulated by depression in patients with movement disorders. Across all subjects, alpha-beta power in the caudate and DLPFC increased during rewarding feedback, while DLPFC alpha-beta power decreased following loss feedback. Reward-related alpha-beta power in both caudate and DLPFC was attenuated in depressed patients compared with nondepressed patients and inversely correlated with depression severity score. Specific to PD, there was also an enhancement of loss-related decrease in DLPFC alpha-beta power in individuals with depression. These results suggest that increased depression symptom severity may correspond with attenuated corticostriatal response to reward and heightened response to loss feedback in patients with PD.

Our findings of reward-related elevations in alpha-beta power are consistent with previous studies examining corticostriatal neural oscillations during cognitive tasks. We previously demonstrated that caudate and DLPFC beta power increases following rewarding feedback in reinforcement learning and working memory tasks (Bick et al., 2019; Paulo et al., 2023). Multiple studies have also previously shown beta power increases associated with reward in corticostriatal and other structures across different cognitive tasks and subject populations (Hosseini and Holroyd, 2015; Andreou et al., 2017; Gueguen et al., 2021; Ricciardi et al., 2023). Increased DLPFC beta power during reward has also been proposed to play a role in coupling attentional and emotional processes during feedback learning and may reflect top-down neural processes in which DLPFC facilitates communication with other brain regions (Cohen et al., 2011; Luft, 2014; Hosseini and Holroyd, 2015; Marco-Pallarés et al., 2015; Li et al., 2017; HajiHosseini et al., 2020). Previous studies have suggested that dorsal striatum neural firing contains information about action value while DLPFC neural firing encodes action selection, suggesting that caudate may provide updated value information to DLPFC to guide action selection (Seo et al., 2012; Bick et al., 2019). The reward-related alpha-beta power elevations we observed in these structures may reflect similar processes.

Importantly, we found that depression symptom severity is correlated with attenuation of reward-related elevations in caudate and DLPFC alpha-beta power. Subjects with depression are known to exhibit dysfunctional behavioral responses to reward, with reduced sensitivity to rewards and maladaptive heightened responses to punishment (Eshel and Roiser, 2010). Previous work has shown that altered fronto-striatal fMRI activation and connectivity during reward processing are prominent neural correlates of depression symptoms (Ubl et al., 2015; Geugies et al., 2022). Individuals with depression have reduced hemodynamic activity in frontal and striatal regions, as well as reduced functional connectivity throughout fronto-striatal circuitry, during reward anticipation and feedback (Robinson et al., 2012; Ubl et al., 2015; Geugies et al., 2022). Our findings suggest that this may be related to altered corticostriatal alpha-beta power changes in response to rewards and losses. These findings are significant because they provide novel information that could be used to guide treatment options such as neuromodulation approaches for treatment-resistant depression in the future.

Compared with nondepressed PD patients, depressed PD patients had attenuated increases in caudate and DLPFC alpha-beta power following reward and greater decreases in DLPFC alpha-beta power following loss feedback. In contrast, DLPFC alpha-beta power did not significantly differ between depressed and nondepressed ET patients following loss feedback. These results suggest that the modulation of DLPFC loss signaling by depression severity that we observed may be specific to PD pathophysiology. Dopaminergic deficits in PD have been shown to correspond with impaired reward processing (Schonberg et al., 2010; Timmer et al., 2017; Costello et al., 2022), and PD patients also have an altered relationship of caudate dopamine and serotonin signaling during reward processing compared with ET patients (Hartle et al., 2025). Alpha power changes related to depressive symptoms in PD have also previously been described in other cortico-striato-thalamo-cortico (CSTC) circuit structures. For example, depressed PD patients have reduced alpha event-related desynchronization in the subthalamic nucleus (STN) in response to positive valence stimuli and increased STN alpha desynchronization in response to negative stimuli (Huebl et al., 2011; Ricciardi et al., 2023). As in our study, these changes in alpha desynchronization were also found to correlate with BDI-II scores (Huebl et al., 2011; Ricciardi et al., 2023). STN has reciprocal connections with the DLPFC. Our work extends these findings into a reward-related context and suggests that they may also be present in other portions of corticostriatal circuitry such as DLPFC. Previous studies have also shown that individuals with depression demonstrate increased learning from loss compared with reward during reward learning, and PD patients have attenuated striatal responses to reward compared with punishment (Timmer et al., 2017). Our findings support these prior conclusions and suggest that alpha-beta power in CSTC circuits could be a biomarker for an enhanced response to loss feedback in PD depression.

Conversely, in ET, literature on the pathophysiology of depression is relatively limited. Our finding that DLPFC alpha-beta power following loss feedback did not significantly differ between depressed and nondepressed ET patients could indicate that the relationship between depression severity and heightened response to loss feedback in corticostriatal structures is unique to PD disease circuitry. ET motor symptoms are associated with dysfunction in cerebellar-thalamic-cortical pathways (Louis, 2010), and studies have suggested that altered cortical-cerebellar connectivity may underlie non-motor symptoms of ET (Duan et al., 2021). Imaging research has also shown that depression symptoms in ET patients correspond with altered functional connectivity in frontal-cerebellar-anterior cingulate circuits, which include the DLPFC, as well as microstructural changes in paralimbic and limbic structures involved in the cerebello-thalamo-cortical pathway (Sengul et al., 2019; Duan et al., 2021).

Limitations of this work include that all participants were undergoing DBS surgery, thus we were unable to compare our dataset to age- and gender-matched healthy control subjects. Additionally, patients generally received light sedation, typically with remifentanil and occasionally with dexmedetomidine or propofol, for their comfort at the start of the surgical procedure. Following the drilling of the burr holes, sedation was stopped to allow the patient to wake up for clinical and research testing. During research testing, patients had generally been off sedating medications for ∼30–40 min. However, potential lingering effects of sedation may have been a confounder for behavioral and neurophysiological results.

The relatively sparse nature of our recordings along clinical DBS trajectories also limited the scope of our neurophysiological analyses, and we did not have sufficient statistical power to perform analyses with hemispheric lateralization. Since only one structure (caudate or DLPFC) was recorded per hemisphere in each patient, we were also unable to perform connectivity analyses between brain regions or with DBS target structures. Additionally, our cortical recordings were all relatively posterior in the DLPFC (Fig. 1). Previous studies have suggested that the DLPFC may have functional subdivisions along the anterior-posterior axis (Koechlin et al., 2003; Jung et al., 2022). Anterior DLPFC subregions have been shown to have greater functional connectivity with frontal and limbic areas as well as the default mode network, while posterior DLPFC subregions show greater functional connectivity with both the default mode network and the multiple demand network, as well as anatomic connections with temporal and parietal areas (Jung et al., 2022). These functional circuits may serve different roles, and imaging research has supported the hypothesis that more anterior subregions of the DLPFC are involved in more abstract cognitive control processing (Koechlin et al., 2003; Jung et al., 2022). Thus, there may have been additional reward and depression-mediated effects on DLPFC signaling that we did not capture from further anterior DLPFC subregions.

Because severe depression is a contraindication to DBS surgery for movement disorders, depressed subjects in our study had mild to moderate depression symptoms. Likewise, the relatively small patient sample size in this study limited the statistical power of some of our comparisons of depression in PD versus ET. Although the proportions of PD and ET patients categorized as depressed in our patient cohort are consistent with depression prevalence across these patient populations and in movement disorder patients undergoing DBS (Louis, 2010; Kratter et al., 2022; Prange et al., 2022; Bishay et al., 2025), no ET patients with caudate recordings in our dataset were categorized as depressed, which limited our disorder-based analysis to the DLPFC. We also assessed depression symptoms and their severity using a self-reported screening measure and not a structured clinical interview, which limits our understanding of history of depression symptoms and comorbid psychiatric conditions.

Overall, this work provides evidence for altered corticostriatal reward-related alpha-beta oscillatory power in depression in two populations of movement disorder patients undergoing DBS. Both PD and ET patients with depression had attenuated DLPFC reward-related alpha-beta power, and depressed PD patients also had reduced caudate reward-related alpha-beta power. Depressed PD patients alone had greater loss-related DLPFC power decrease. These findings have important implications for guiding future therapeutic developments for treatment-resistant depression (TRD) as well as the comorbid depression symptoms of PD and ET. DBS, for instance, has generated significant interest as a treatment option for severe TRD. Likewise, left DLPFC is a common target for transcranial magnetic stimulation for TRD. Our findings implicate alpha-beta power attenuation within corticostriatal circuits as a potential therapeutic target for adaptive stimulation techniques to modulate altered reward signaling in depression.

## Synthesis

Reviewing Editor: Laura Volpicelli-Daley, The University of Alabama at Birmingham Decisions are customarily a result of the Reviewing Editor and the peer reviewers coming together and discussing their recommendations until a consensus is reached. When revisions are invited, a fact-based synthesis statement explaining their decision and outlining what is needed to prepare a revision will be listed below. The following reviewer(s) agreed to reveal their identity: Nicole Bentley.

The findings are the manuscript are of interest to the neuroscience community and add nuance to our understanding of reward circuitry in this population. The data analysis is reasonable, though a connectivity analysis between caudate/DLPFC/targets of DBS would increase the impact of the findings. The main critique is that there is no mention of where the recordings are from within DLPFC and there is no information on hemispheric lateralization. Further comments/questions are outlined below.

1. L 113. Please provide more detail to clarify the recording approach. Was the cannula held in place at the surface with the macro-contact just beneath the surface?

2. L 129: Regarding the task, how long did the task take to complete?

3. L 129: Also regarding the task, were patients given sedation that could confound results?

4. L 256-262: I was confused about why PD results are given here in this section pertaining to ET. If these are in reference to PD, it seems out of place. Was it meant to reference ET?

5. L 335-337: Would caution against over-interpreting results. Reward circuitry and attention control may be (likely are) very different.

6. L. 349: Please clarify what kind of "activity" is reduced

7. L. 364: Please define opponency signaling.

8. Overall, these results are of interest, but DLPFC is a large region with heterogeneous organization. Please provide a schematic or more detailed description of where recordings occurred and how the particular sub-regional constraint of DLPFC might affect the findings. The sparse nature of these recordings sites should also be mentioned in the limitations.
