# Supplementary material for: Movement Disorder Patients with Depression Have Altered Corticostriatal Alpha-Beta Power Response to Reward and Loss
Source: eNeuro. 2026 Jul 9;13(7):ENEURO.0008-26.2026. doi: 10.1523/ENEURO.0008-26.2026 (PMC13364504; doi:10.1523/ENEURO.0008-26.2026)
Supplement: Table 1-1 — Patient demographic and disease-related information. DLPFC = dorsolateral prefrontal cortex, ET = essential tremor, GPI = globus pallidus internus, PD = Parkinson’s disease, STN = subthalamic nucleus, VIM = ventral intermediate nucleus of the thalamus. Download Table 1-1, DOCX file. [file eneuro-13-ENEURO.0008-26.2026-s002.docx]

**Extended Data Table 1-1. Patient demographic and disease-related information.**

| **Subject ID** | **Disorder** | **Target (Bilateral)** | **Age (Years)** | **Sex** | **BDI-II** | **Depressed** | **% Trials Correct** | **Caudate Channels** | **DLPFC Channels** | |  |
| --- | --- | --- | --- | --- | --- | --- | --- | --- | --- | --- | --- |
| 1 | PD | STN | 50s | M | 21 | Yes | 79.17 | 2 | 3 | |  |
| 2 | PD | STN | 60s | M | 4 | No | 87.67 | 3 | | 2 | |
| 3 | PD | STN | 60s | F | 0 | No | 52.74 | 3 | | 3 | |
| 4 | ET | VIM | 40s | F | 2 | No | 77.4 | 2 | | 2 | |
| 5 | ET | VIM | 70s | F | 7 | No | 54.79 | 1 | | 2 | |
| 6 | ET | VIM | 70s | M | 10 | No | 52.05 | 0 | | 2 | |
| 7 | PD | STN | 50s | M | 19 | Yes | 74.07 | 0 | | 6 | |
| 8 | PD | STN | 60s | M | 12 | No | 87.67 | 2 | | 3 | |
| 9 | ET | VIM | 60s | M | 7 | No | 88.36 | 4 | | 0 | |
| 10 | ET | VIM | 60s | F | 16 | Yes | 76.79 | 0 | | 4 | |
| 11 | PD | STN | 50s | M | 8 | No | 84.93 | 3 | | 3 | |
| 12 | ET | VIM | 60s | F | 2 | No | 71.23 | 0 | | 2 | |
| 13 | PD | STN | 60s | M | 4 | No | 72.6 | 2 | | 3 | |
| 14 | ET | VIM | 70s | M | 9 | No | 82.19 | 2 | | 1 | |
| 15 | PD | STN | 60s | M | 20 | Yes | 84.25 | 3 | | 0 | |
| 16 | PD | GPI | 50s | M | 7 | No | 84.93 | 0 | | 6 | |
| 17 | PD | GPI | 50s | M | 9 | No | 78.77 | 0 | | 5 | |
| 18 | PD | GPI | 70s | M | 18 | Yes | 49.32 | 2 | | 3 | |
| 19 | ET | VIM | 70s | F | 8 | No | 79.45 | 1 | | 0 | |
| 20 | PD | STN | 60s | M | 22 | Yes | 78.77 | 2 | | 3 | |
| 21 | ET | VIM | 60s | M | 7 | No | 73.97 | 0 | | 4 | |
| 22 | ET | VIM | 50s | M | 24 | Yes | 63.7 | 0 | | 2 | |
| 23 | ET | VIM | 60s | M | 10 | No | 78.77 | 0 | | 4 | |
| 24 | PD | GPI | 70s | F | 3 | No | 65.75 | 0 | | 6 | |
| 25 | PD | GPI | 60s | M | 9 | No | 79.17 | 1 | | 3 | |
| 26 | ET | VIM | 50s | M | 1 | No | 70.83 | 1 | | 2 | |
| 27 | ET | VIM | 70s | M | 5 | No | 68.75 | 2 | | 2 | |
| 28 | ET | VIM | 70s | F | 2 | No | 77.08 | 2 | | 0 | |
| 29 | ET | VIM | 60s | M | 0 | No | 87.5 | 1 | | 2 | |
| 30 | ET | VIM | 40s | M | 9 | No | 52.08 | 1 | | 1 | |
| **Total / Average ± SD** | **14 PD, 16 ET** | **9 STN, 5 GPI, 16 VIM** | **63.5 ± 8.85** | **22 M, 8 F** | **9.17 ± 6.93** | **7 Yes, 23 No** | **73.83 ± 11.69** | **40** | | **79** | |

DLPFC = dorsolateral prefrontal cortex, ET = essential tremor, GPI = globus pallidus internus, PD = Parkinson’s disease, STN = subthalamic nucleus, VIM = ventral intermediate nucleus of the thalamus.
