# Supplementary material for: Movement Disorder Patients with Depression Have Altered Corticostriatal Alpha-Beta Power Response to Reward and Loss
Source: eNeuro. 2026 Jul 9;13(7):ENEURO.0008-26.2026. doi: 10.1523/ENEURO.0008-26.2026 (PMC13364504; doi:10.1523/ENEURO.0008-26.2026)
Supplement: Figure 6-1 — Caudate channels significantly involved in reward signaling. PD = Parkinson’s disease, ET = essential tremor. Download Figure 6-1, DOCX file. [file eneuro-13-ENEURO.0008-26.2026-s003.docx]

**Extended Data Figure 6-1. Caudate channels significantly involved in reward signaling.**

| **Subject ID** | **Significant Channels** | **Disorder** | **BDI-II** | **Depression Status** | **Comparison** |
| --- | --- | --- | --- | --- | --- |
| 3 | 3 | PD | 0 | Non-depressed | Correct vs Baseline |
| 8 | 2 | PD | 12 | Non-depressed | Correct vs Baseline |
| 11 | 3 | PD | 8 | Non-depressed | Correct vs Baseline |
| 13 | 1 | PD | 4 | Non-depressed | Correct vs Baseline |
| 25 | 1 | PD | 9 | Non-depressed | Correct vs Baseline |
| 9 | 4 | ET | 7 | Non-depressed | Correct vs Baseline |
| 14 | 2 | ET | 9 | Non-depressed | Correct vs Baseline |
| 26 | 1 | ET | 1 | Non-depressed | Correct vs Baseline |
| 29 | 1 | ET | 0 | Non-depressed | Correct vs Baseline |
| 30 | 1 | ET | 9 | Non-depressed | Correct vs Baseline |
| 2 | 3 | PD | 4 | Non-depressed | Incorrect vs Baseline |
| 18 | 2 | PD | 18 | Depressed | Incorrect vs Baseline |
| 20 | 2 | PD | 22 | Depressed | Incorrect vs Baseline |
| 5 | 1 | ET | 7 | Non-depressed | Incorrect vs Baseline |
| 14 | 1 | ET | 9 | Non-depressed | Incorrect vs Baseline |
| **Total / Average** | 19 Channels | 5 PD, 5 ET | 5.9 Average | 0 Depressed | Correct vs Baseline |
|  | 9 Channels | 2 PD, 2 ET | 12 Average | 2 Depressed | Incorrect vs Baseline |

PD = Parkinson’s disease, ET = essential tremor.
