# Supplementary material for: Movement Disorder Patients with Depression Have Altered Corticostriatal Alpha-Beta Power Response to Reward and Loss
Source: eNeuro. 2026 Jul 9;13(7):ENEURO.0008-26.2026. doi: 10.1523/ENEURO.0008-26.2026 (PMC13364504; doi:10.1523/ENEURO.0008-26.2026)
Supplement: Figure 6-2 — DLPFC channels significantly involved in reward signaling. DLPFC = dorsolateral prefrontal cortex, PD = Parkinson’s disease, ET = essential tremor. Download Figure 6-2, DOCX file. [file eneuro-13-ENEURO.0008-26.2026-s004.docx]

**Extended Data Figure 6-2. DLPFC channels significantly involved in reward signaling.**

| **Subject ID** | **Significant Channels** | **Disorder** | **BDI-II** | **Depression Status** | **Comparison** |
| --- | --- | --- | --- | --- | --- |
| 2 | 1 | PD | 4 | Non-depressed | Correct vs Baseline |
| 3 | 3 | PD | 0 | Non-depressed | Correct vs Baseline |
| 7 | 1 | PD | 19 | Depressed | Correct vs Baseline |
| 8 | 3 | PD | 12 | Non-depressed | Correct vs Baseline |
| 11 | 3 | PD | 8 | Non-depressed | Correct vs Baseline |
| 13 | 2 | PD | 4 | Non-depressed | Correct vs Baseline |
| 17 | 2 | PD | 9 | Non-depressed | Correct vs Baseline |
| 25 | 3 | PD | 9 | Non-depressed | Correct vs Baseline |
| 4 | 1 | ET | 2 | Non-depressed | Correct vs Baseline |
| 23 | 1 | ET | 10 | Non-depressed | Correct vs Baseline |
| 26 | 2 | ET | 1 | Non-depressed | Correct vs Baseline |
| 2 | 2 | PD | 4 | Non-depressed | Incorrect vs Baseline |
| 8 | 1 | PD | 12 | Non-depressed | Incorrect vs Baseline |
| 20 | 3 | PD | 22 | Depressed | Incorrect vs Baseline |
| 14 | 1 | ET | 9 | Non-depressed | Incorrect vs Baseline |
| 21 | 2 | ET | 7 | Non-depressed | Incorrect vs Baseline |
| 22 | 2 | ET | 24 | Depressed | Incorrect vs Baseline |
| 23 | 2 | ET | 10 | Non-depressed | Incorrect vs Baseline |
| **Total / Average** | 22 Channels | 8 PD, 3 ET | 7.1 Average | 1 Depressed | Correct vs Baseline |
|  | 13 Channels | 3 PD, 4 ET | 12.6 Average | 2 Depressed | Incorrect vs Baseline |

DLPFC = dorsolateral prefrontal cortex, PD = Parkinson’s disease, ET = essential tremor.
