# Supplementary material for: Movement Disorder Patients with Depression Have Altered Corticostriatal Alpha-Beta Power Response to Reward and Loss
Source: eNeuro. 2026 Jul 9;13(7):ENEURO.0008-26.2026. doi: 10.1523/ENEURO.0008-26.2026 (PMC13364504; doi:10.1523/ENEURO.0008-26.2026)
Supplement: Figure 7-1 — Linear mixed effects model results for caudate alpha-beta power during correct trials. DF = degrees of freedom, CI = confidence interval. Download Figure 7-1, DOCX file. [file eneuro-13-ENEURO.0008-26.2026-s005.docx]

**Extended Data Figure 7-1. Linear mixed effects model results for caudate alpha-beta power during correct trials.**

| **Predictor** | **Estimate** | **Standard Error** | **t-Value** | **DF** | **p_corr_** | **95% CI Lower Bound** | **95% CI Upper Bound** |
| --- | --- | --- | --- | --- | --- | --- | --- |
| **BDI-II** | -0.014 | 0.0031 | -4.6 | 36 | 1.2E-04 | -0.02 | -0.0078 |
| **Movement Disorder** | -0.11 | 0.070 | -1.5 | 36 | 0.28 | -0.25 | 0.036 |
| **BDI-II*Movement Disorder** | 0.022 | 0.0099 | 2.3 | 36 | 0.061 | 0.0022 | 0.042 |

DF = degrees of freedom, CI = confidence interval.
