# Supplementary material for: Movement Disorder Patients with Depression Have Altered Corticostriatal Alpha-Beta Power Response to Reward and Loss
Source: eNeuro. 2026 Jul 9;13(7):ENEURO.0008-26.2026. doi: 10.1523/ENEURO.0008-26.2026 (PMC13364504; doi:10.1523/ENEURO.0008-26.2026)
Supplement: Figure 7-2 — Linear mixed effects model results for DLPFC alpha-beta power during correct trials. DF = degrees of freedom, CI = confidence interval. Download Figure 7-2, DOCX file. [file eneuro-13-ENEURO.0008-26.2026-s006.docx]

**Extended Data Figure 7-2. Linear mixed effects model results for DLPFC alpha-beta power during correct trials.**

| **Predictor** | **Estimate** | **Standard Error** | **t-Value** | **DF** | **p_corr_** | **95% CI Lower Bound** | **95% CI Upper Bound** |
| --- | --- | --- | --- | --- | --- | --- | --- |
| **BDI-II** | -0.0075 | 0.0023 | -3.2 | 75 | 3.5E-03 | -0.012 | -0.0029 |
| **Movement Disorder** | -0.084 | 0.045 | -1.9 | 75 | 0.12 | -0.17 | 0.0044 |
| **BDI-II*Movement Disorder** | 0.0033 | 0.0040 | 0.83 | 75 | 0.81 | -0.0046 | 0.011 |

DF = degrees of freedom, CI = confidence interval.
