# Supplementary material for: Movement Disorder Patients with Depression Have Altered Corticostriatal Alpha-Beta Power Response to Reward and Loss
Source: eNeuro. 2026 Jul 9;13(7):ENEURO.0008-26.2026. doi: 10.1523/ENEURO.0008-26.2026 (PMC13364504; doi:10.1523/ENEURO.0008-26.2026)
Supplement: Figure 7-3 — Linear mixed effects model results for caudate alpha-beta power during incorrect trials. DF = degrees of freedom, CI = confidence interval. Download Figure 7-3, DOCX file. [file eneuro-13-ENEURO.0008-26.2026-s007.docx]

**Extended Data Figure 7-3. Linear mixed effects model results for caudate alpha-beta power during incorrect trials.**

| **Predictor** | **Estimate** | **Standard Error** | **t-Value** | **DF** | **p_corr_** | **95% CI Lower Bound** | **95% CI Upper Bound** |
| --- | --- | --- | --- | --- | --- | --- | --- |
| **BDI-II** | -0.0083 | 0.0031 | -2.7 | 36 | 2.2E-02 | -0.015 | -0.002 |
| **Movement Disorder** | -0.019 | 0.071 | -0.27 | 36 | 1.6 | -0.16 | 0.12 |
| **BDI-II*Movement Disorder** | 0.0028 | 0.0099 | 0.28 | 36 | 1.6 | -0.017 | 0.023 |

DF = degrees of freedom, CI = confidence interval.
