# Supplementary material for: Movement Disorder Patients with Depression Have Altered Corticostriatal Alpha-Beta Power Response to Reward and Loss
Source: eNeuro. 2026 Jul 9;13(7):ENEURO.0008-26.2026. doi: 10.1523/ENEURO.0008-26.2026 (PMC13364504; doi:10.1523/ENEURO.0008-26.2026)
Supplement: Figure 7-4 — Linear mixed effects model results for DLPFC alpha-beta power during incorrect trials. DF = degrees of freedom, CI = confidence interval. Download Figure 7-4, DOCX file. [file eneuro-13-ENEURO.0008-26.2026-s008.docx]

**Extended Data Figure 7-4. Linear mixed effects model results for DLPFC alpha-beta power during incorrect trials.**

| **Predictor** | **Estimate** | **Standard Error** | **t-Value** | **DF** | **p_corr_** | **95% CI Lower Bound** | **95% CI Upper Bound** |
| --- | --- | --- | --- | --- | --- | --- | --- |
| **BDI-II** | -0.0076 | 0.0018 | -4.3 | 75 | 1.1E-04 | -0.011 | -0.0041 |
| **Movement Disorder** | -0.15 | 0.034 | -4.3 | 75 | 1.1E-04 | -0.22 | -0.079 |
| **BDI-II*Movement Disorder** | 0.011 | 0.0031 | 3.7 | 75 | 0.00082 | 0.0052 | 0.017 |

DF = degrees of freedom, CI = confidence interval.
